# FACTORS ASSOCIATED WITH ELDER MISTREATMENT AMONG PERSONS LIVING WITH DEMENTIA

**DOI:** 10.1093/geroni/igad104.0607

**Published:** 2023-12-21

**Authors:** Zach Gassoumis, Jeanine Yonashiro-Cho, Elizabeth Avent, Laura Mosqueda

**Affiliations:** University of Southern California, Los Angeles, California, United States; University of Southern California, Los Angeles, California, United States; University of Southern California, Los Angeles, California, United States; Keck School of Medicine of the University of Southern California, Los Angeles, California, United States

## Abstract

Persons living with dementia (PLWD) are at increased risk of experiencing elder mistreatment (EM), including emotional, physical, or sexual abuse, neglect, or financial exploitation. Because PLWD rely on Care Partners (CP) for varying degrees of support with ADLs/IADLs, factors contributing to EM occurrence may be present in the characteristics of the PLWD, CP, and the environment in which they interact. The present study sought to identify associated risk and protective factors of EM in PLWD-CP care groups. As part of a longitudinal study, survey administration interviews were conducted with 163 care groups consisting of a PLWD and their CP(s) (n=184) to gather data on participant characteristics, current and past relationship quality, current caregiving interactions, and EM occurrence. This analysis used baseline data with responses weighted to control for participation of multiple CPs. Bivariate logistic regression was used to model the likelihood of the PLWD experiencing EM. Nearly a quarter of PLWDs (23.9%; n=39) experienced EM, with emotional abuse (19.0%), neglect (7.4%), and financial exploitation (4.3%) occurring most frequently. Preliminary analyses reveal significant bivariate associations between EM and PLWD alcohol/substance abuse (OR=3.244;CI=1.020-10.320) and educational attainment (OR=1.495;CI=1.077-2.075), and CP symptoms of depression (OR=1.170;CI=1.046-1.308) or anxiety (OR=1.194;CI=1.085-1.315), high caregiving burden (OR=1.099;CI=1.042-1.159), expectations of themselves (OR=1.747;CI=1.064-2.867) and the PLWD (OR=1.730;CI=1.113-2.688), and alcohol/substance abuse (OR=2.163;CI=1.080-4.333). These associations provide a foundation for further evaluation of these characteristics as potential longitudinal risk factors for EM, and provide potential targets for the development of prevention and intervention strategies that are tailored to care groups’ characteristics and needs.

